# Local delivery of dinutuximab from lyophilized silk fibroin foams for treatment of an orthotopic neuroblastoma model

**DOI:** 10.1002/cam4.2936

**Published:** 2020-02-24

**Authors:** Kimberly J. Ornell, Jordan S. Taylor, Jasmine Zeki, Naohiko Ikegaki, Hiroyuki Shimada, Jeannine M. Coburn, Bill Chiu

**Affiliations:** ^1^ Department of Biomedical Engineering Worcester Polytechnic Institute Worcester MA USA; ^2^ Department of Surgery Division of Pediatric Surgery Stanford University Stanford CA USA; ^3^ Department of Surgery Division of Pediatric Surgery University of Illinois at Chicago Chicago IL USA; ^4^ Department of Anatomy and Cell Biology University of Illinois at Chicago Chicago IL USA; ^5^ Department of Pathology and Laboratory Medicine University of Southern California Los Angeles CA USA

**Keywords:** Ch14.18, dinutuximab, immunotherapy, local delivery, neuroblastoma, silk fibroin

## Abstract

Immunotherapy targeting GD2 is a primary treatment for patients with high‐risk neuroblastoma. Dinutuximab is a monoclonal antibody with great clinical promise but is limited by side effects such as severe pain. Local delivery has emerged as a potential mechanism to deliver higher doses of therapeutics into the tumor bed, while limiting systemic toxicity. We aim to deliver dinutuximab locally in a lyophilized silk fibroin foam for the treatment of an orthotopic neuroblastoma mouse model. Dinutuximab‐loaded silk fibroin foams were fabricated through lyophilization. In vitro release profile and bioactivity of the release through complement‐dependent cytotoxicity were characterized. MYCN‐amplified neuroblastoma cells (KELLY) were injected into the left gland of mice to generate an orthotopic neuroblastoma model. Once the tumor volume reached 100 mm^3^, dinutuximab‐, human IgG‐, or buffer‐loaded foams were implanted into the tumor and growth was monitored using high‐resolution ultrasound. Post‐resection histology was performed on tumors. Dinutuximab‐loaded silk fibroin foams exhibited a burst release, with slow release thereafter in vitro with maintenance of bioactivity. The dinutuximab‐loaded foam significantly inhibited xenograft tumor growth compared to IgG‐ and buffer‐loaded foams. Histological analysis revealed the presence of dinutuximab within the tumor and neutrophils and macrophages infiltrating into dinutuximab‐loaded silk foam. Tumors treated with local dinutuximab had decreased MYCN expression on histology compared to control or IgG‐treated tumors. Silk fibroin foams offer a mechanism for local release of dinutuximab within the neuroblastoma tumor. This local delivery achieved a significant decrease in tumor growth rate in a mouse orthotopic tumor model.

## Graphical abstract

Dinutuximab, an immunotherapy targeting GD2, was delivered loacally in a lyophilized silk fibroin foam for the treatment of an orthotopic neuroblastoma mouse model. Silk fibroin foam allows for a sustained release of dinutuximab, causing significant tumor growth inhibition.

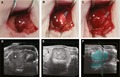

## 1 INTRODUCTION

Neuroblastoma is one of the most common solid tumors affecting children, accounting for approximately 15% of all childhood cancer deaths.^1^ It accounts for 6% of all childhood cancers and is the most common cancer in infants under 1 year of age.^1^ Nearly half of all patients are classified as having a high‐risk disease, portending poor long‐term survival.^2^ Treatment for neuroblastoma consists of a multimodal treatment approach including surgical resection, chemotherapy and radiation. Immunotherapy has emerged as a promising adjuvant therapy to improve outcomes in patients with high‐risk neuroblastoma.^3^ GD2 is a disialoganglioside and promising tumor antigen present in both neuroblastoma and melanoma. In noncancerous tissue, GD2 expression is limited to the peripheral sensory nerves and melanocytes.^4^, ^5^ This restricted expression in noncancerous tissue coupled with expression across almost all neuroblastoma cells makes antibodies targeting GD2 highly suitable for immunotherapy.^6^

Dinutuximab (ch14.18) is a chimeric monoclonal antibody targeting GD2, consisting of an Fc portion of a human IgG1 immunoglobulin fused with the Fab portion of a murine 14G2a antibody.^7^ Mujoo et al^8^ in 1987 showed that the systemic administration of ch14.18 via intraperitoneal route slowed the growth of human SKNAS neuroblastoma xenografts established at subcutaneous sites. More recent preclinical studies demonstrated that ch14.18 induces antibody‐dependent cell‐mediated cytotoxicity and complement‐dependent cytotoxicity (CDC) in human melanoma and neuroblastoma.^9^, ^10^ Ch14.18 has shown promise in early clinical trials as a single agent.^4^ In a phase I clinical trial, nine pediatric patients received ch14.18 and five showed either a mixed or partial response.^11^ In an additional clinical study, ch14.18 was evaluated on high‐risk neuroblastoma patients during the maintenance phase of their treatment. As compared to maintenance chemotherapy or no further treatment, a higher overall survival was exhibited in patients receiving ch14.18.^12^ This activity was shown to be enhanced when ch14.18 was combined with either granulocyte‐macrophage colony‐stimulating factor (GM‐CSF) or interleukin‐2 (IL‐2).^6^

In a combination clinical trial, Yu et al^6^ examined the efficacy of immunotherapy with dinutuximab in patients with high‐risk neuroblastoma who had responded to induction therapy and stem cell transplant. They demonstrated that dinutuximab in combination with GM‐CSF and IL‐2 was superior to standard therapy with regards to rates of event‐free survival (65% vs 46%, *P* = .02) and overall survival (86% vs 75%, *P* = .02) at two years. Although administering dinutuximab in combination with GM‐CSF and IL‐2 is feasible in patients with neuroblastoma after autologous bone marrow transplantation or stem cell rescue,^13^ there are toxic effects associated with systemic immunotherapy. Yu et al^6^ attributed these to antibody binding to GD2 expressed on normal nerve cells causing pain in more than 50% of patients, capillary leak and hypersensitivity reactions. Furthermore, additional clinical trials both with dinutuximab as a single agent and in combination with immunocytokines have cited pain as the most common side effect, in some cases even precluding the use of higher ch14.18 doses.^14^

Local drug delivery is emerging as an alternative to the traditional systemic delivery of therapeutics for the management of solid tumors.^15^ Local administration of therapeutic agents can offer significant benefits to patients, including reducing the frequency and severity of toxic side effects associated with systemic therapies.^16^ Clinically, local chemotherapy delivery systems, such as the Gliadel^®^ Wafer, have been employed for the treatment of solid tumors.^17^ In neuroblastoma models, other local delivery methods have been employed, delivering high concentrations of various chemotherapy agents in a targeted fashion, effectively slowing tumor growth while reducing systemic side effects.^18^, ^19^, ^20^

Silk fibroin is a promising biomaterial for delivery platforms due to its structure, ability to be processed into multiple formats (gels, films, fibers, foams, etc), and stability at high temperatures and in a range of pH.^21^ Clinically, silk fibroin has not only been used as suture material, but has also been used in implantable scaffolds.^22^ As a drug delivery platform, silk fibroin has been shown to cause minimal immune response in vivo,^23^, ^24^, ^25^, ^26^, ^27^, ^28^ while allowing for local and sustained release of various chemotherapy agents.^18^, ^19^, ^20^, ^29^ Furthermore, lyophilized silk hydrogel systems have shown great promise for local sustained release of monoclonal antibodies.^30^

We hypothesized that lyophilization of dinutuximab with silk fibroin into an implantable foam can be used to create a sustained release platform for local dinutuximab delivery. Furthermore, we hypothesized that local delivery of dinutuximab via silk fibroin foam would lead to slowed tumor growth in neuroblastoma tumor.

## 2 MATERIALS AND METHODS

### 2.1 Antibodies

The mouse‐human chimeric anti‐GD2 antibody, ch14.18 was provided by United Therapeutics. The control purified IgG from human plasma was purchased from Innovative Research.

### 2.2 Silk fibroin extraction

Silk fibroin from *Bombxy mori* silkworm cocoons (Tajima Shoji Co.), kindly provided by Dr David L. Kaplan at Tufts University, was extracted as described previously.^21^ Briefly, five grams of cocoons were cut into approximately 1 cm^2^ pieces and boiled in 0.02 mol/L Na_2_CO_3_ for 30 minutes to extract the sericin. The silk fibroin fibers were dried overnight and then dissolved in 9.3 mol/L LiBr at 60°C for 3 hours. The dissolved silk fibroin was dialyzed against ultrapure water for two days with at least six water changes in 3500 MWCO dialysis tubing (Fisher Scientific). The aqueous silk fibroin (referred to as silk from here on) solution was stored at 4°C for future use.

### 2.3 Silk foam fabrication

**Figure 1:**
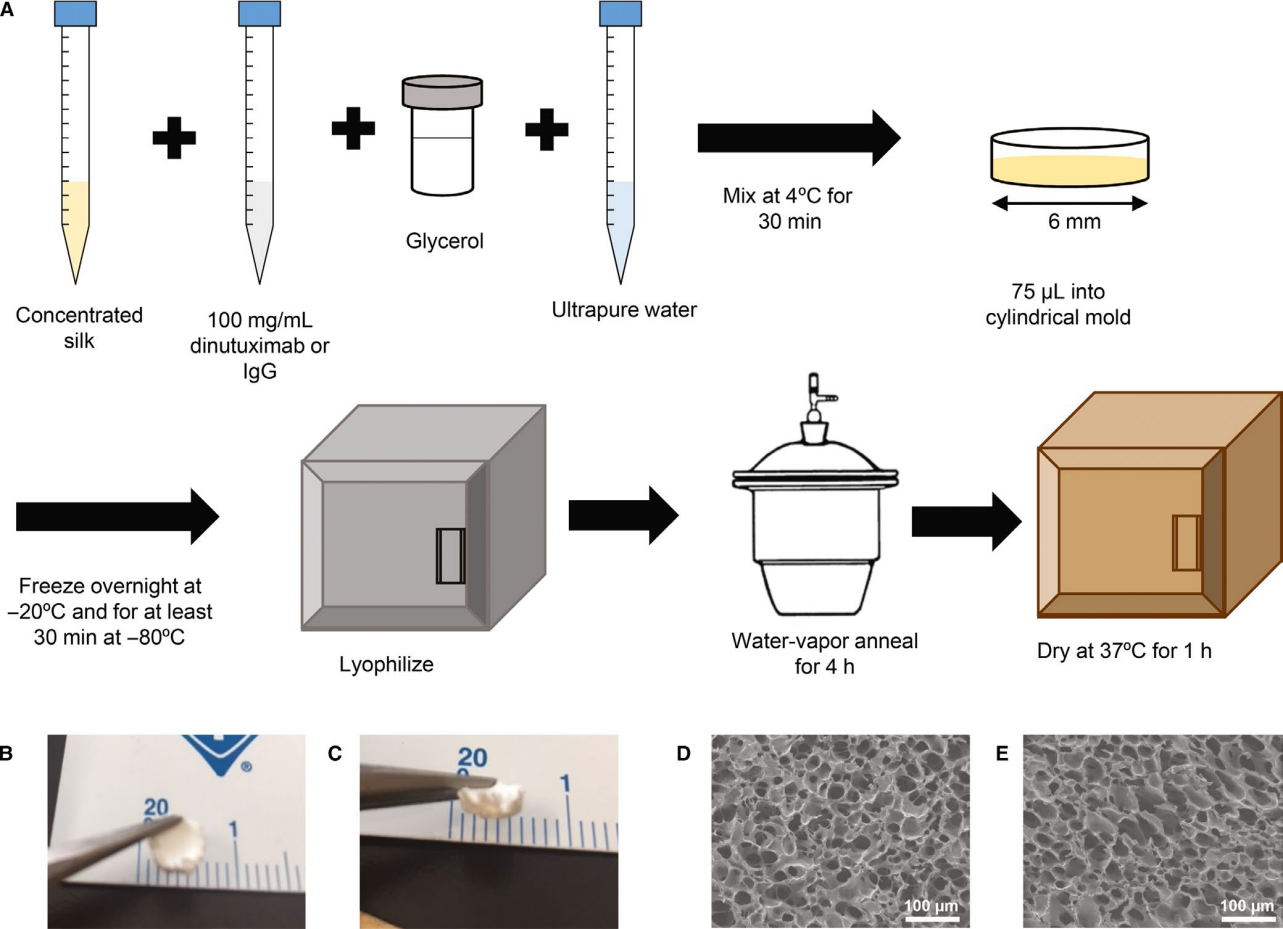
Schematic for fabricating dinutuximab‐loaded silk foams. (A) Concentrated silk is mixed with dinutuximab, glycerol, and water, followed by freezing and lyophilizing; water‐vapor annealing is used to induce crystallinity followed by sample drying. Post‐lyophilization, the samples retain their shape and size for both (B) dinutuximab‐ and (C) IgG‐loading. The ultrastructure of the lyophilized materials as seen by SEM for (D) dinutuximab‐loaded foams and (E) IgG‐loaded foams. SEM, scanning electron microscopy

Silk, concentrated dinutuximab (United Therapeutics), and glycerol were mixed to yield a final concentration of 10% (w/v) silk, 26.7 mg/mL dinutuximab, and 25% (w/silk weight) glycerol. For control foams, dinutuximab was replaced with Protein A Purified human IgG (Innovative Research), or the equivalent volume of antibody solution replaced with buffer (20 mmol/L histidine, 0.05% polysorbate 20, 150 mmol/L sodium chloride). Samples were fabricated in cylindrical molds (six mm diameter) using a volume of 75 µL. The samples were frozen at −20°C overnight, transferred to a −80°C freezer for at least of 30 minutes, and lyophilized. To induce the β‐sheet confirmation of the silk fibroin, thereby altering the material crystallinity and rendering the material insoluble, the lyophilized samples were water‐vapor annealed in the molds for 4 hours and dried at 37°C for 1 hour (Figure 1).^28^

### 2.4 Scanning electron microscopy

The morphology of silk foams fabricated with dinutuximab or IgG was visualized using scanning electron microscopy. Samples were hydrated briefly, cut to expose the cross section of the foam, and dried overnight. The dry samples were sputter coated with gold (25 mA, 60 seconds) and imaged with a field emission scanning electron microscope using a three kV electron beam (JEOL 7000F, JEOL).

### 2.5 Dinutuximab and IgG release

Dinutuximab‐, IgG‐, or buffer‐loaded silk foams were placed in 1.7 mL protein lo‐bind tubes in 1.2 mL of PBS at 37°C. At varying time points, one mL of PBS was removed and replaced with fresh PBS (Figure S1). The protein release concentration over the first 24 hours was measured via reading the absorbance at 280 nm (Nanodrop 2000, ThermoFisher). The absorbance of the buffer‐loaded silk controls was used as a background control (Table S1). After 24 hours of release, the protein concentration was determined using an enzyme‐linked immunosorbent assay. Briefly, release samples were incubated in 96‐well plates (Grenier Bio‐One) for 1 hour at 37°C. The plates were then washed with PBS containing 0.05% Tween‐20. HRP‐conjugated goat anti‐human IgG Fc (Jackson Labs) was added at 40 ng/well in PBS containing 4.5% non‐fat milk and, 0.05% Tween‐20 and incubated for 30 minutes at 37°C. The plate was washed with PBS containing 0.05% Tween‐20 and 50 µL of TMB substrate (BioFX, VWR) was added. After 15 minutes, 50 µL of two N sulfuric acid was added to stop the reaction. The absorbance was read at 450 nm using a SpectraMax 250 microplate reader (Molecular Devices). Buffer‐loaded silk controls were analyzed to confirm that silk or buffer did not contribute to the calculated dinutuximab concentration.

### 2.6 Cell culture

KELLY neuroblastoma cells (Millipore Sigma) were maintained in Roswell Park Memorial Institute 1640 (RPMI) Medium supplemented with 10% (v/v) fetal bovine serum, 100 U/mL penicillin, 100 µg/mL streptomycin, and two mM L‐glutamine (Fisher Scientific). All cells were maintained at 37°C at 5% CO_2_ in a humidified environment. Cells were passaged using 0.25% trypsin‐EDTA at 70%‐80% confluence.

### 2.7 Fluorescent activated cell sorting

KELLY cells, grown as described above, were made into a single cell suspension and washed twice with PBS. Cells were incubated with primary antibody at a concentration of 1 µg/1 × 10^6^ cells in 100 µL for 1 hour at 4°C. Cells were then washed in PBS supplemented with 100 U/mL penicillin, 100 µg/mL streptomycin. AlexFluor^®^488‐conjugated goat anti‐human IgG secondary antibody (Jackson Laboratories) was added to the cells at 1:500 dilution and incubated for 40 minutes at 4°C. Cells were then resuspended in phenol red free media. Just prior to sorting, cells were stained with 7AAD (TONBO Biosciences) at a concentration of 3 µL/mL. The KELLY cells that expressed the highest GD2 (top 12%) were collected. Cells were sorted at the University of Massachusetts Medical Center Flow Cytometry Core using an Aria II cell sorter (BD Biosciences).

### 2.8 Complement‐dependent cytotoxicity assay

KELLY cells were made into a single cell suspension and stained in 10 µM calcein (Thermo Fisher) at a concentration of 1 × 10^6^ cells/mL for 30 minutes at 37°C. Cells were washed twice in PBS and separated into aliquots of 5 × 10^4^ cells in 80 µL of complete RPMI media. To determine the toxicity of human serum with active complement, complement was added giving a final concentration of 10% (v/v). Samples from the release study or stock dinutuximab (20 µL) were added and incubated for 15 minutes at room temperature. Controls included cells not exposed to antibody (negative control) and cells treated with 20 µL of 2% Triton™ X‐100 (positive control). The cells were incubated at room temperature, protected from light for 2 hours to allow for complement‐dependent cell death to initiate. After 2 hours, the cell suspension was centrifuged and the amount of calcein released in the supernatant was quantified using a Victor^3^ Multilabel Reader (Perkin Elmer) at an excitation of 485 nm and an emission of 535 nm. All data were background subtracted using the negative control and normalized to the signal obtained by the positive control.

### 2.9 Mouse orthotopic neuroblastoma model

All mouse procedures were performed in accordance with the National Institute of Health protocols on Humane Care and Use of Laboratory Animals and approved by the Institutional Animal Care and Use Committee at University of Illinois at Chicago. Seven‐week old female NCr nude mice (Harlan) were used to create an orthotopic neuroblastoma tumor model as previously described.^16^, ^18^, ^19^ In brief, the mice were anesthetized with inhaled isoflurane and an incision was made on the left flank to locate the adrenal gland. KELLY cells (1 × 10^6^ in two µL of PBS) were injected into the adrenal gland and the incision was closed in layers. Tumor growth was followed using a VisualSonics Vevo 2100 sonographic probe (Toronto) as described previously.^19^ In brief, serial cross‐sectional images of the left adrenal and tumor were obtained (0.076 mm between images). Tumor volumes were measured by a single observer (JZ) to limit variability, using a 3‐D reconstruction tool (Vevo Software v1.6.0). When tumor volume reached 100 mm^3^, mice underwent repeat operation in order to implant the silk foam within the tumor. The previous flank incision was opened and the tumor capsule was incised with electrocautery so the foam could be placed within the tumor. The facial and skin incisions were closed in layers and the tumor growth was again monitored with high frequency ultrasound. Once tumor volume exceeded 1000 mm^3^, mice were sacrificed and tumor specimens were processed for histology. Animal weights were recorded twice weekly as a surrogate for systemic toxicity.

### 2.10 Histology

Tumor specimens adjacent to control, IgG‐loaded, and dinutuximab‐loaded foam were obtained for hematoxylin and eosin (H&E) staining. Specimens were fixed in 10% buffered formalin, serially dehydrated and embedded in paraffin. Tumor specimens were sectioned (5 µm thickness) and affixed to glass slides for H&E staining. H&E sections were reviewed and annotated by a histopathologist with expertise in neuroblastoma (HS).

For GD2 staining and determining the presence of dinutuximab within the tumors, tumors were frozen without fixation in OCT compound and cryosectioned at a thickness of 10 µm. Sections were blocked for endogenous peroxidases with 0.3% H_2_O_2_, washed and fixed with 4% paraformaldehyde. Sections were then blocked in 5% normal goat serum (NGS, ThermoFisher) in PBS. For staining of GD2, sections were incubated with dinutuximab at a concentration of 10 µg/mL overnight at 4°C in PBS with 1% NGS. Sections were washed and treated with goat anti‐human IgG conjugated with HRP (Jackson Labs) for 2 hours. Staining for residual dinutuximab or IgG was performed by exposing sections to secondary antibody without primary antibody. Detection of HRP was performed using a DAB substrate system (VWR). Sections were counterstained with Myers hematoxylin and washed in Scott's Tap Water.

Finally, additional sections were stained with anti‐MYCN mouse monoclonal antibody (NCM II 100) as described previously in order to determine level of MYCN expression in the control, IgG‐, and dinutuximab‐treated tumors.^31^, ^32^

### 2.11 Tumor dissociation

After euthanizing and sterilizing the mouse, a midline incision was made, and the tumor was excised. The tumor was cut into small pieces, placed into a solution of RPMI media and collagenase (type IV) and incubated at 37°C for a minimum of 3 hours. After incubation, the solution was passed through a cell strainer (40 µm). Tumor cells were isolated using lymphocyte separation medium (Corning^®^) and washed several times with serum free RPMI1640. Cells were then frozen in donor equine serum containing 10% DMSO.

### 2.12 Flow cytometry on dissociated tumor cells

Frozen tumor cells (as dissociated above) were made into a single cell suspension and washed with PBS. Cells were incubated with dinutuximab at a concentration of 1 µg/1 × 10^6^ cells in 100 µL for 1 hour at 4°C. Cells were then washed in PBS supplemented with 1% FBS and 0.02% sodium azide at a concentration of 1 × 10^6^ cells/mL. Secondary antibody (AlexaFluor^®^488‐conjugated goat anti‐human IgG, Jackson Laboratories) was added to the cells at 1:1000 dilution and incubated for 40 minutes at 4°C. Cells were then washed twice with PBS, fixed for 10 minutes in 4% paraformaldehyde, and washed again with PBS. Cells were suspended at of 1 × 10^6^ cells in 100 µL. Flow cytometry was performed on an Accuri 6 Flow cytometer (BD Biosciences). Analysis was performed using FlowJo^®^ software with a minimum of 5000 events captured for each sample.

### 2.13 Statistical analysis

Statistics were performed using GraphPad Prism 5. For direct comparisons of two groups, an unpaired students *t* test was used, with a significance value of *P* < .05. For comparisons across multiple groups, a one‐way analysis of variance (ANOVA) test with a boneferoni correction and Tukey post‐hoc tests for pairwise comparisons between groups.

## 3 RESULTS

### 3.1 Silk foam formation and morphology

Silk films containing dinutuximab or IgG were fabricated by lyophilizing a mixture of antibody and silk, with glycerol to stabilize the foams (Figure 1A). During this process ice crystals form while freezing and sublimed during lyophilization which created pores in place of the ice crystals, thus creating a porous foam (Figure 1B‐E). Microscale pores within the foams can be observed through scanning electron microscope of the interior of the foam (Figure 1D,E). No differences in morphology were observed between the pore structure of dinutuximab and IgG loaded foams.

### 3.2 Dinutuximab and IgG in vitro release

**Figure 2:**
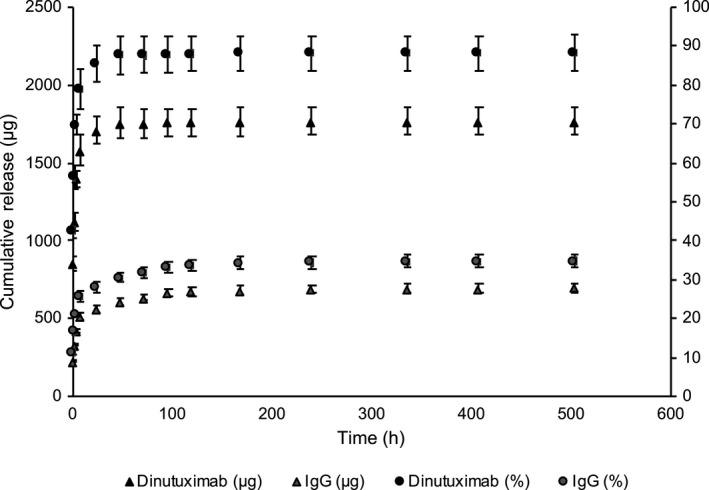
In vitro release profile of dinutuximab‐ and IgG‐loaded silk foams. In vitro release of dinutuximab and IgG shown as cumulative percent of dinutuximab and IgG released relative to the initial 2000 µg loaded into each foam and mass of dinutuximab and IgG released through the entire time period

The release of dinutuximab and IgG from lyophilized silk foams was measured in vitro (Figure 2). The burst release in the first 24 hours was approximately 85% of the loaded antibody for the dinutuximab and 28% of the loaded antibody for the IgG. Over the next 14 days, approximately 3% of the loaded dinutuximab was released and 7% of the IgG was released. The average release rate for the dinutuximab after the first 24 hours for the subsequent six days was nine µg/day. After the first week, the release of dinutuximab slowed but sustained a release at approximately 0.1 µg/day from 7‐14 days, and 0.03 µg/day for 14‐21 days. The average release rate for the IgG sample after the first 24 hours for the subsequent 6 days was 12 µg/day. After the first week, the release of IgG slowed to 1.4 µg/day from 7 to 14 days, and 0.3 µg/day from 14 to 21 days. Interestingly, a much larger burst release was observed for the dinutuximab as compared to the IgG. After 21 days, nearly complete release of dinutuximab was observed (89%), while only 35% of IgG was released (Figure 2).

### 3.3 Evaluation of in vitro dinutuximab activity

Dinutuximab is an immunotherapeutic that has been shown to have several mechanisms of action against cancer cells. One such mechanism is CDC.^33^ In CDC, the complement protein C1q binds to the IgG domain of an antibody that displays CDC activity leading to the formation of a membrane attach complex on the target cells, resulting in cell lysis.

**Figure 3:**
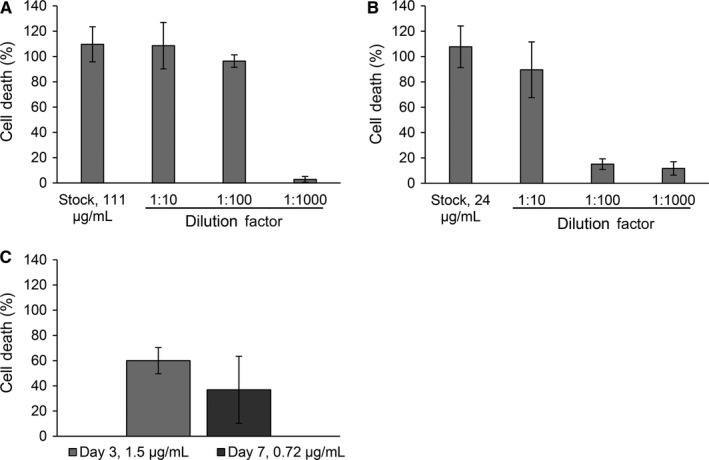
Complement‐dependent cytotoxicity of release product in vitro. complement‐dependent cytotoxicity performed on high‐GD2‐expressing KELLY cells with release product from in vitro release study. Cytotoxicity is expressed as percent of dead cells as compared to maximal cell lysis. complement‐dependent cytotoxicity is induced by release product from (A) 0‐1 hours, (B) 8‐24 hours, and (C) up to day seven of release. Values for concentration shown were obtained from the NanoDrop or the ELISA measurements. ELISA, enzyme‐linked immunosorbent assay

In vitro, antibody‐induced CDC can be readily assayed using cells labeled with calcein AM, a molecule that becomes fluorescent upon internalization by cells and is retained within the viable cells.^34^ When the cells undergo lysis, the fluorescent calcein is released and readily detected in the cell culture media.^34^ Here, the released dinutuximab was evaluated using this CDC assay to determine if dinutuximab remained active during the fabrication and antibody release process using high‐GD2‐expressing KELLY neuroblastoma cells collected using fluorescent activated cell sorting (FACS) (Figure S2). To ensure that the complement serum was not causing excess toxicity to KELLY cells, cells treated with only 10% complement (no antibody) were compared to cells not treated with antibody or complement serum. No statistical difference was observed between cells exposed to no treatment and cells exposed to only 10% complement (Figure S3). The range of non‐released dinutuximab activity can be detected between 1.1 and 0.1 µg/mL (data not shown). The amount of cell death induced by the release product collected at different time intervals was quantified (Figure 3). At early time points, the release product induced nearly complete cell death compared to the positive control (Figure 3A,B). At early time points, the release product was well above the range of detection for the assay (1.1 µg/mL vs 111 µg/mL at 0‐1 hour, and 24 µg/mL at 8‐24 hours). In order to evaluate the effectiveness of the release product at concentrations within the range of detection, the release product was diluted and evaluated for toxicity. Antibody‐induced CDC was observed in samples collected as far as 14 days into the release study (Figure 3C). Time points later than 14 days fell below the effective range of the assay. This suggests that the dinutuximab released (within the effective range) remained in its active form and induced toxicity to KELLY cells. To confirm that no cell death was induced by human IgG (control antibody), the CDC assay was performed with IgG at concentrations of 0.5, 1 mg/mL, and from the 1 hour release time point, as well as dinutuximab at a concentration of 0.1 mg/mL. Cell lysis comparable to that of the positive control (0.3% Triton™ X‐100) was observed in the cells treated with dinutuximab, while cell death comparable with that of the 10% complement or no treatment condition was observed with all the IgG conditions (Figure S3).

### 3.4 Effect of dinutuximab‐loaded silk foams on orthotopic neuroblastoma tumor growth

**Figure 4:**
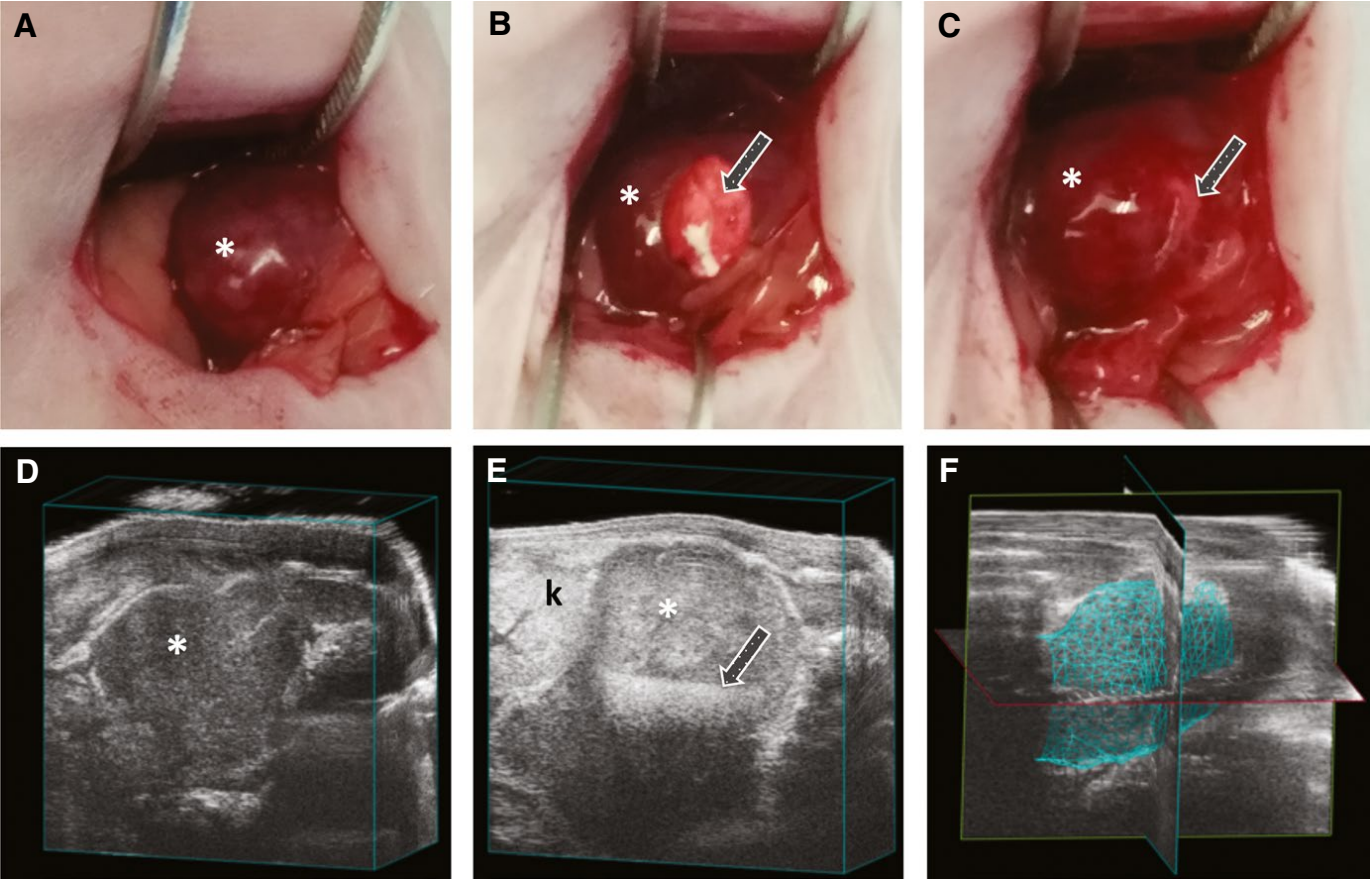
Implantation of silk foam into orthotopic neuroblastoma and ultrasound imaging. Orthotopic neuroblastoma (A) just prior to implantation of silk foam, (B) with silk foam halfway implanted and (C) completely implanted. Tumor growth/volume was followed with high‐frequency ultrasound (D) before and (E) after treatment with silk foam; (F) representative 3D image of tumor used to obtain volumetric measurements. Asterisk (*) indicates tumor location; arrow (→) denotes position of silk foam within the tumor; k indicates the left kidney

**Figure 5:**
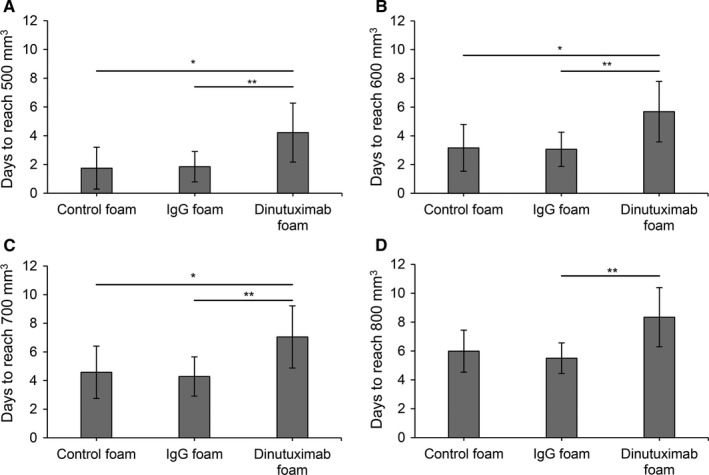
Tumor response to treatment. Days for tumors in each treatment group (buffer‐, IgG‐, or dinutuximab‐loaded foam) to reach (A) 500 mm^3^, (B) 600 mm^3^, (C) 700 mm^3^, or (D) 800 mm^3^ after implantation, *P < .05, **P < .01, ***P < .001

To determine the in vivo efficacy of the dinutuximab release system, xenograft studies were performed using a mouse orthotopic neuroblastoma model using the high‐GD2 expressing KELLY neuroblastoma cells. Once the orthotopic neuroblastoma tumors reached 100 mm^3^ by ultrasound, mice underwent implantation of silk foam that was dinutuximab‐loaded, human IgG‐loaded (antibody control), or buffer‐loaded (negative control) (Figure 4). There was no difference in initial tumor size between the various groups. Tumors treated with dinutuximab‐loaded silk foam reached 500 mm^3^ after 4.22 ± 2.05 days, significantly slower than tumors treated with buffer‐loaded silk foam (1.74 ± 1.46 days, *P* = .02) or IgG‐loaded foam (1.85 ± 1.06 days, *P* = .009) (Figure 5A). Similarly, tumors treated with dinutuximab‐loaded silk foam reached 600, 700, and 800 mm^3^ significantly slower than either IgG‐ or buffer‐loaded silk foam treated tumors (Figure 5B‐D). There was no significant difference in the timing to reach 500, 600, 700, or 800 mm^3^ between tumors treated with buffer‐ or IgG‐loaded silk foam (*P* = .9, .9, .7, and .6, respectively). Dinutuximab‐loaded silk foam treatment was more effective at decreasing tumor growth compared to IgG‐ or buffer loaded‐foam. Finally, there was no significant difference in animal weights across the treatment groups.

### 3.5 Histological evaluation of tumors

**Figure 6:**
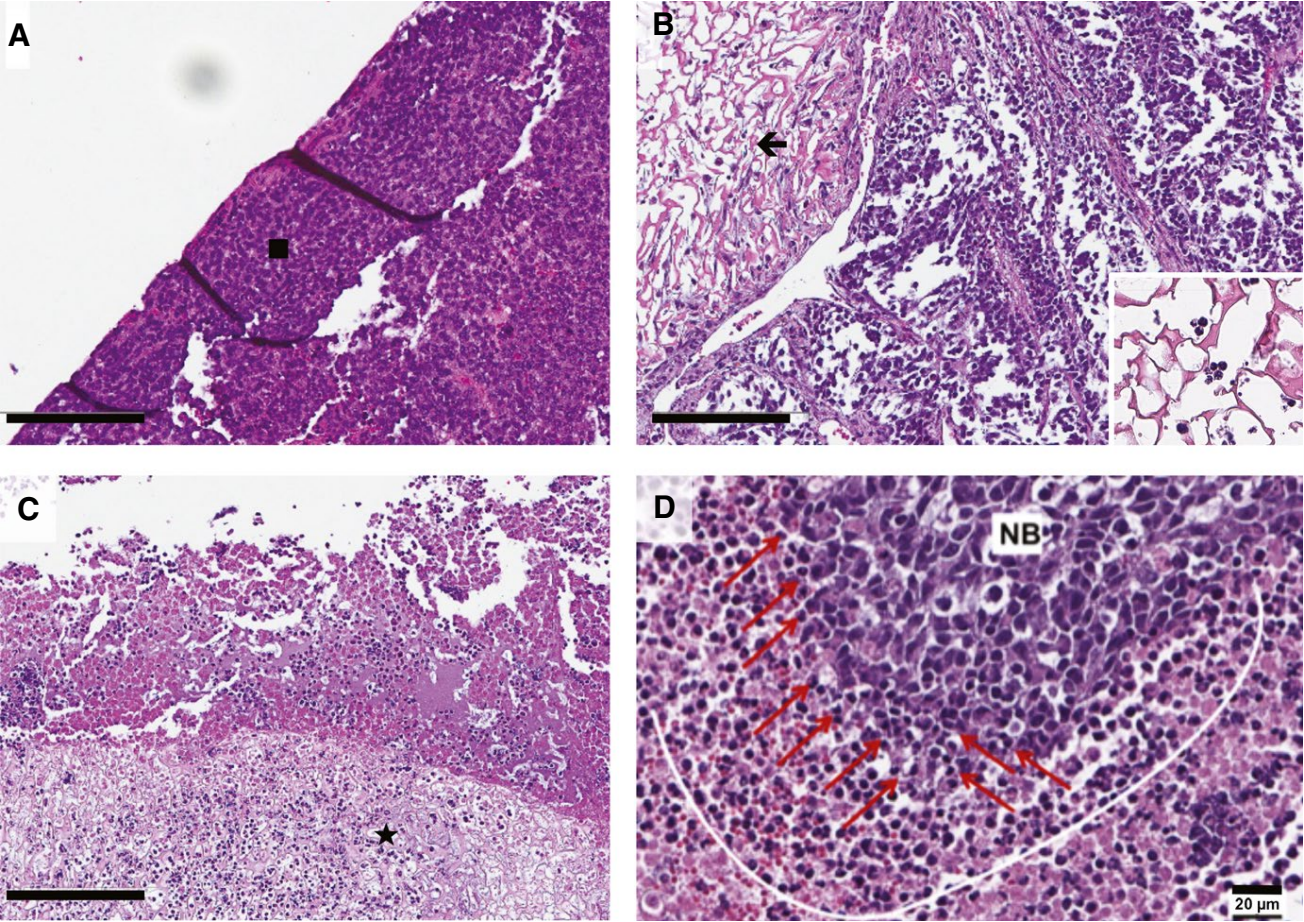
Hematoxylin and eosin stains of paraffin‐embedded tumor sections. A, Control foam treated tumor; square denotes viable neuroblastoma with classic small round blue cells; B, IgG‐loaded foam treated tumor; black arrow (←) denotes silk foam structure adjacent the tumor; insert depicts higher magnification of silk foam without significant infiltration of neutrophils or macrophages; C, dinutuximab‐loaded foam treated tumor; star denotes the silk foam adjacent the tumor, with infiltrating neutrophils and macrophages, scale bar for A‐C represents 200 µm; D, Higher magnification of dinutuximab‐treated tumor, with a layer of neutrophils and macrophages (outlined by thin white line) formed around neuroblastoma (NB); those cells directly involved in cytotoxic activity are indicated by the red arrows 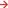 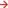; scale bar represents 20 µm

Regardless of treatment group, mice were sacrificed when tumor volume reached 1000 mm^3^. Tumors were formalin fixed and embedded in paraffin. Tumor sections adjacent silk foam were stained with H&E and examined (Figure 6). Tumors treated with dinutuximab‐loaded foam showed increased infiltration of macrophages and neutrophils into the foam and adjacent tumor (Figure 6C) relative to the buffer or IgG‐treated tumor sections (Figure 6A,B).

### 3.6 GD2 status and dinutuximab retention within the tumor bed

**Figure 7:**
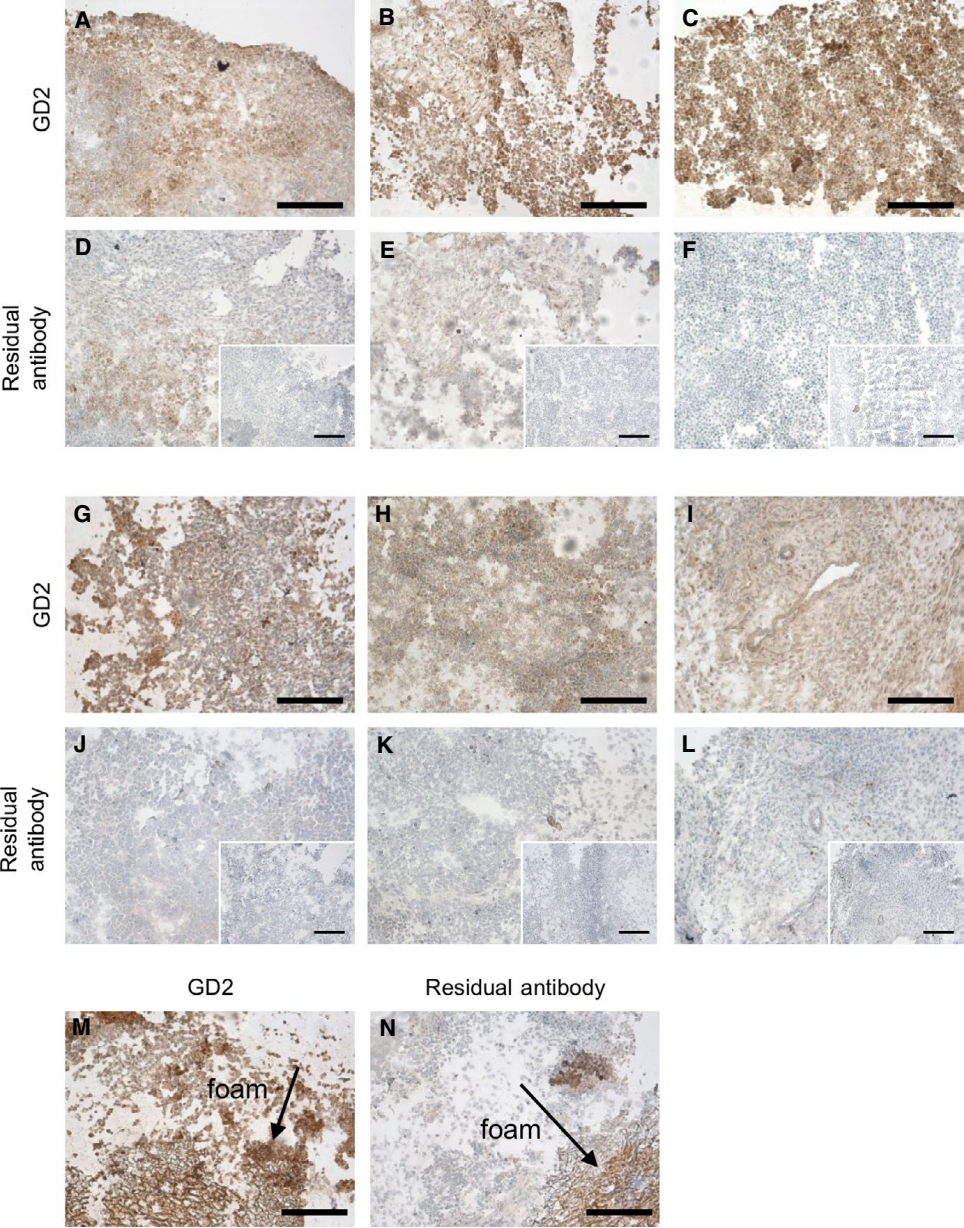
Evaluation of treated tumors for GD2 expression and residual antibody. Immunohistochemistry was performed to evaluate GD2 expression and residual antibody in treated tumors. Tumors which were allowed to grow for six or less days after exposure to foams containing (A, D) dinutuximab, (B, E) IgG, or (C, F) no antibody (control) and more than six days after exposure to foams containing (D, J) dinutuximab, (E, K) IgG, or (F, L) no antibody (control) were evaluated. Staining and imaging (A‐C and G‐I) confirmed retention of GD2 expression throughout the tumors. Residual (D) dinutuximab and (IgG) can be observed within the tumor body in the earlier time point specimens. Image inserts represent negative controls, tumor slides not exposed to antibodies. The foam could be visualized within the tumor body with positive staining for (M) GD2 expression and (N) residual dinutuximab, even in long‐term samples

To confirm GD2 status and presence of residual antibody in the tumor tissue, immunohistochemistry was performed on excised tumors. Previous clinical data has shown that treatment with monoclonal antibodies does not result in a loss of GD2 expression in neuroblastoma tumors.^35^ Consistent with this, we see no loss in GD2 expression in tumors regardless of exposure to dinutuximab‐loaded foams for a short treatment window less than six days, Figure 7A‐C. Further, when comparing tumors exposed to dinutuximab‐loaded foams for a longer treatment window (greater than six days), GD2 expression remained unchanged (Figure 7G‐H). Additional sections were exposed to anti‐IgG antibody to determine if dinutuximab or IgG remained in the tumor tissue following treatment (Figure 7D‐F,J,K). Anti‐IgG binding was visualized by a low degree of positive staining, as compared to sections exposed to only DAB substrate (inlayed images in Figure 7D‐F,J,K). In tumors exposed to dinutuximab‐loaded foams for a longer treatment window (greater than six days) the foams continued to be visualized adjacent to the tumor bed (Figure 7M,N). These data support retention of antibody in the tumor tissue.

### 3.7 MYCN status of tumor after treatment

**Figure 8:**
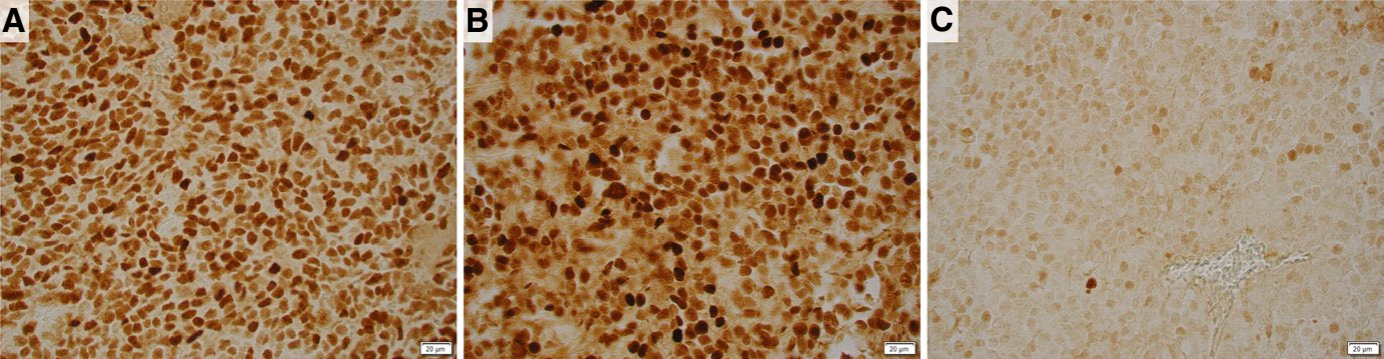
MYCN expression in orthotopic KELLY tumors after local treatment. Anti‐MYCN antibody demonstrates positive staining on (A) control and (B) IgG‐treated tumors; however, (C) there is a significant loss of MYCN expression in the dinutuximab‐treated tumors; scale bar represents 20 µm

Orthotopic KELLY tumor sections were stained with anti‐MCYN antibody after treatment with either control, IgG‐, or dinutuximab‐loaded silk foam. KELLY tumors treated with control silk foam were distinctly positive for anti‐MYCN staining on immunohistochemistry (Figure 8A). IgG‐treated tumors were similarly positive for MYCN. MCYN expression in KELLY tumors treated with dinutuximab‐loaded silk foam; however, was significantly decreased compared to both control or IgG‐treated tumors (Figure 8C).

### 3.8 Flow cytometry analysis of dinutuximab‐, IgG‐, and buffer‐loaded foam‐treated orthotopic neuroblastoma tumors

To validate the above histology staining performed on tumors treated with dinutuximab‐, IgG‐, or buffer‐loaded foams, flow cytometry was performed on GD2 expression of the dissociated tumor cells. Consistent with both the histology and previous clinical data,^33^ there was no decrease in GD2 expression in the treated tumors (Figure S4). As compared to the secondary only controls, all cells stained positive for GD2.

## 4 DISCUSSION

Dinutuximab is a promising therapeutic for neuroblastoma as it targets the GD2 disialoganglioside, which is highly expressed on tumor and has restricted expression on normal tissue. In both preclinical and clinical trials, the chimeric human‐murine antibody ch14.18 (dinutuximab) has shown promise in elimination of neuroblastoma cells through antibody‐dependent cellular cytotoxicity and complement‐dependent cellular cytotoxicity.^8^, ^10^, ^36^, ^37^ However, the larger protein size results in poor tumor penetration and the antibody binds to peripheral nerves, causing patients a great deal of pain.^36^ This is the limiting toxicity factor in many patients. One potential method to increase tumor penetration of antibody and reduce side effects in patients is local delivery, which has previously been demonstrated to result in higher intra‐tumoral drug concentration and consistently low systemic concentration when compared with administering the drug systemically.^18^ Combining silk and dinutuximab in an aqueous system and lyophilizing it has the potential to create a local release system for focal dinutuximab therapy. In these experiments, we demonstrated that local treatment with dinutuximab can decrease orthotopic tumor growth and MYCN expression in a MYCN‐amplified tumor. The decrease in MYCN expression after dinutuximab treatment is significant, and may explain one mechanism behind the clinical benefit observed from using anti‐GD2 immunotherapy.^38^ Future studies will need to investigate the significance of this effect, particularly examining how dinutuximab or anti‐GD2 therapy could change the risk profile of MYCN‐amplified neuroblastoma. The differential tumor growth demonstrates that dinutuximab delivered locally improves local‐regional control; however, the effects this treatment might have on metastatic disease are still unknown. The model used in these experiments could be used to investigate these effects, as it has high fidelity to high risk disease, with neuroblastoma cells resideing in the adrenal gland,^19^ and the presence of metastasis in bone marrow as demonstrated by immunohistochemical markers such as Phox2B (data not shown).

In this study, lyophilized silk foams were fabricated to evaluate the impact of local delivery of dinutuximab in an orthotopic neuroblastoma model. Lyophilized silk foams are advantageous as they are robust enough to be handled with forceps while allowing a flexible architecture for implantation, and results in a dry product that is better equipped for long‐term storage. In addition, previous work with silk fibroin has demonstrated that there is a limited macrophage response to extracted silk fibroin fibers as determined by cytokine secretion and transcript level.^25^ The immune response observed in non‐processed silk fibers (such as sutures) has been demonstrated to be due to the sericin proteins that coat the native silk.^25^ In long term studies, silk has been demonstrated to be well tolerated with less pronounced immune responses than materials such as PLA, PVA, and collagen.^39^, ^40^, ^41^ Furthermore, the fabrication from an all aqueous process eliminates any need for harmful solvents and allows for control over properties such as the crystallinity and, in the case of antibody release, the amount of molecular entrapment.^42^ Previous work performed by Guziewicz et al,^30^ showed that lyophilization of silk hydrogels allows for sustained release of biologically active monoclonal antibodies. Additionally, lyophilized silk has been shown to have a stabilizing effect on enzymes and other macromolecules, which is expected to extend to antibodies.^43^ The silk‐based platform for local delivery also offers the advantage of tailored release profile based on fabrication and distribution of treatment within the tumor bed.^44^, ^45^

Silk fibroin is also a versatile substrate that can be fabricated into numerous forms suitable for various clinical situations. We have previously demonstrated that silk fibroin can be loaded with chemotherapy in an injectable gel form and can be administered locally via intra‐tumoral injections.^18^, ^45^, ^46^ This therapy could be administered at various stages of disease treatment: Initially, it could be administered via image‐guided (ultrasound or computed tomography scan) injection to complement other systemic therapy. It could also be utilized in cases of aggressive tumors at the time of resection, when a complete resection is not feasible or is too risky. The drug‐loaded silk material could be applied to the post‐resection tumor bed.^19^, ^20^ The various formats of the silk material such as film or sponge could be modified so that drug‐loaded silk material could be applied to the tumor through a trocar in a minimally invasive laparoscopically assisted procedure.

The use of immunocompromised mice is common practice for studying oncology therapeutics. The athymic NCr nude mice have deficient T cell function, which allows them to grow xenografts using human malignant cell lines. However, these mice retain a similar number of B cells, natural killer cells, neutrophils, and macrophages,^47^, ^48^, ^49^ allowing them to have a response to treatment even with immunotherapy agents. As discussed previously, the efficacy of dinutuximab as an antitumor therapy is based on the antibody‐mediated and compliment‐mediated cytotoxicity.^9^, ^10^ Therefore, the NCr nude mice xenograft models are appropriate for the study of dinutuximab.

The engineered silk foams can deliver an initial (burst) and slow release of antibody over time. We hypothesize that the mechanisms of release are through the diffusion through the silk‐phase of the silk foams, hydrophobic/hydrophilic interactions, and hydration resistance to the silk foams, as has previously been demonstrated with lyophilized silk hydrogels loaded with antibody.^30^ Of note, the difference between the release profiles for dinutuximab and IgG could be attributed to the different salt compositions in the buffer from which the lyophilized antibody preparations were prepared. Additionally, differences in release could be due to the structural differences between mouse‐human chimeric dinutuximab and human IgG impacting the hydrophilic/hydrophobic interactions. Future studies could examine the effect of salt compositions and concentrations on release profiles of antibodies form silk hydrogels.

Previous studies have shown that greater than 90% of KELLY cells are positive for GD2.^43^ However, within this positive population there remains heterogeneous expression of GD2 (Figure S2B). Clinically, the majority of neuroblastoma tumor cells exhibit GD2 on the cell membrane making it a prominent therapeutic target.^50^ Furthermore, high expression of GD2 is one of the hall marks of high‐risk neuroblastoma.^51^ Therefore, by isolating the high expressing GD2 cells, we aimed to best mimic the target population of cells within the tumor.

One of the most important criteria of a local release system is the preservation of bioactivity of the release product. We have evaluated the bioactivity of released dinutuximab in vitro using a CDC assay on Kelly cells with high GD2 expression. CDC has previously been identified as one of the driving mechanisms of dinutuximab toxicity. CDC occurs when an antibody binds to its target cells in the presence of active complement proteins. The complement protein, specifically Cq1, binds to the antibody and begins a cascade that results in cell lysis through a membrane attack complex.^52^ While the complement cascade does differ between human and mice, studies with a similarly structured chimeric antibody Rituximab (targeted to CD20) have shown that the complement cascade is both active and necessary in a mouse model.^53^, ^54^, ^55^ This suggests that CDC is a relevant in vitro assay to measure bioactivity and can be translated in vivo, even in an immunocompromised mouse model. The potential CDC response coupled with the retention of natural killer cells, neutrophils, and macrophages in this mouse model allowing for antibody‐dependent cell mediated cytoxicity is likely driving the significant decrease in tumor growth rate.^47^, ^48^, ^49^

## 5 CONCLUSION

Our data suggest that lyophilized silk foams can deliver active dinutuximab to the tumor tissue locally with burst and slow release thereafter. The delivery of dinutuximab in this fashion significantly reduced growth of KELLY cell xenografts in comparison to human IgG and buffer only controls in an orthotopic tumor model. Future studies can evaluate alterations in dinutuximab side effects, particularly pain from local delivery as compared to traditional intravenous delivery. Additionally, more work is needed to understand how the decrease in MYCN expression seen after local dinutuximab therapy might contribute to improved tumor response.

## CONFLICTS OF INTEREST

The authors have no conflicts of interest to declare related to this work.

## AUTHOR CONTRIBUTIONS

Drs. Kimberly J. Ornell, Bill Chiu, Naohiko Ikegaki, and Jeannine M. Coburn conceptualized and designed the study, aided in acquiring and analyzing data, drafted and critically revised the manuscript. Ms Zeki helped aquire, analyze, and interpret the data, and was involved with drafting the manuscript. Dr Taylor aided in analyzing and interpreting the data, drafting the manuscript, and making critical revisions to the final manuscript. Dr Shimada was involved in study design, analyzing and interpreting the data, and critically revised the manuscript.

## Data Availability

The data that support the findings of this study are available from the corresponding author upon reasonable request.
