# Supplementary material for: Local delivery of dinutuximab from lyophilized silk fibroin foams for treatment of an orthotopic neuroblastoma model
Source: Cancer Med. 2020 Feb 24;9(8):2891–903. doi: 10.1002/cam4.2936 (PMC7163090; doi:10.1002/cam4.2936)
Supplement: Supplementary file 1 [file CAM4-9-2891-s001.pdf]

SUPPLEMENTAL INFORMATION - ***Local delivery of dinutuximab from lyophilized silk fibroin foams for treatment of an orthotopic neuroblastoma model.*** **Ornell K, Taylor JS, Zeki J et al.**

Supplemental Figure 1

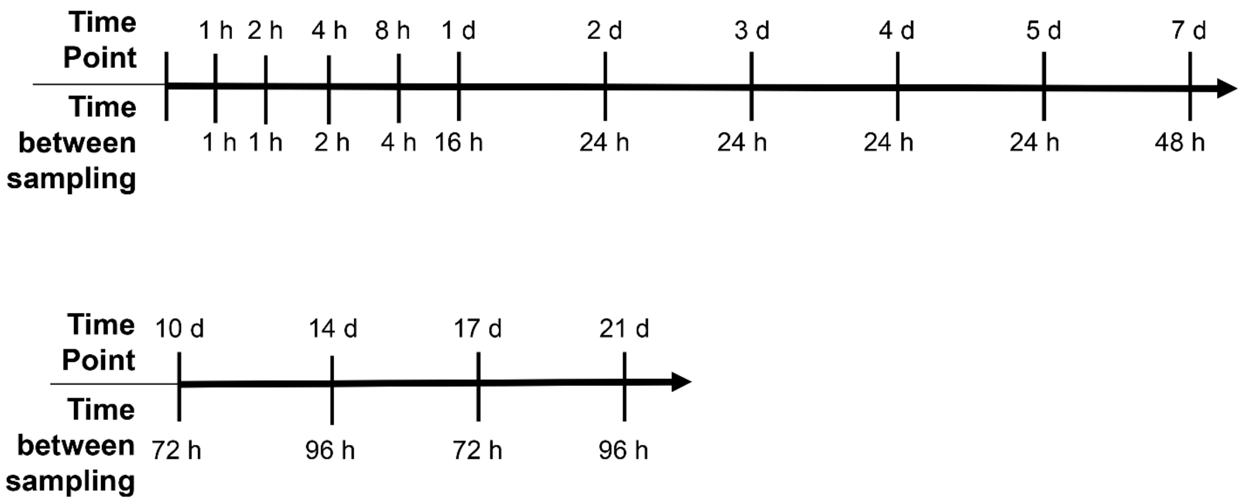

Figure S1. Experimental design for release studies. On day 0, 1.2 mL of PBS was added to a protein low-bind 1.7 mL tube containing a scaffold loaded with dinutuximab, IgG, or buffer. At each time point, one mL of solution was removed and replaced with one mL of fresh PBS.

Supplemental Table 1

A

A280 Readings mg/mL (2 readings were taken from each sample)

Sample	1 h	2 h	4 h	8 h	24 h
**Control 1**	0.041	-0.037	0.015	-0.005	-0.014
**Control 1**	0.074	-0.055	0	-0.002	-0.011
**Control 2**	0.075	0.014	0	-0.011	-0.003
**Control 2**	0.054	0.004	0.004	0.005	0.016
**Control 3**	0.049	0.003	-0.017	-0.008	0.001
**Control 3**	0.059	0.009	0	-0.021	0.007
**Control 4**	0.072	0.01	0.008	0.006	-0.004
**Control 4**	0.023	0.007	0.01	-0.007	-0.006

B

	48 h	96 h	168 h	336h	504 h
**ELISA Readings for silk + buffer**	-0.0045	-0.0055	-0.0055	-0.0055	-0.0065

ELISA Standard Curve

Concentration (ng/mL)	Blank	0.034	0.095	0.265	0.741	2.075
	-0.0055	0.0175	0.0595	0.1695	0.4525	0.9275
	0.0055	0.0185	0.0605	0.1545	0.4225	0.8695

Table S1. Dinutuximab release by absorbance and ELISA. In order to standardize the release profile, a control sample with silk and buffer only was run alongside the dinutuximab-loaded silk. For the A280 data, the buffer-loaded silk control was subtracted from the dinutuximab loaded silk samples. (A) The A280 readings of silk for the first 24 hours are provided here. In addition, to confirm silk was not contributing to the release in the ELISA assay, undiluted samples from the buffer-loaded control were analyzed. (B) The absorbance values as compared to the blank and the low end of the dinutuximab standard curve are provided here.

Supplemental Figure 2

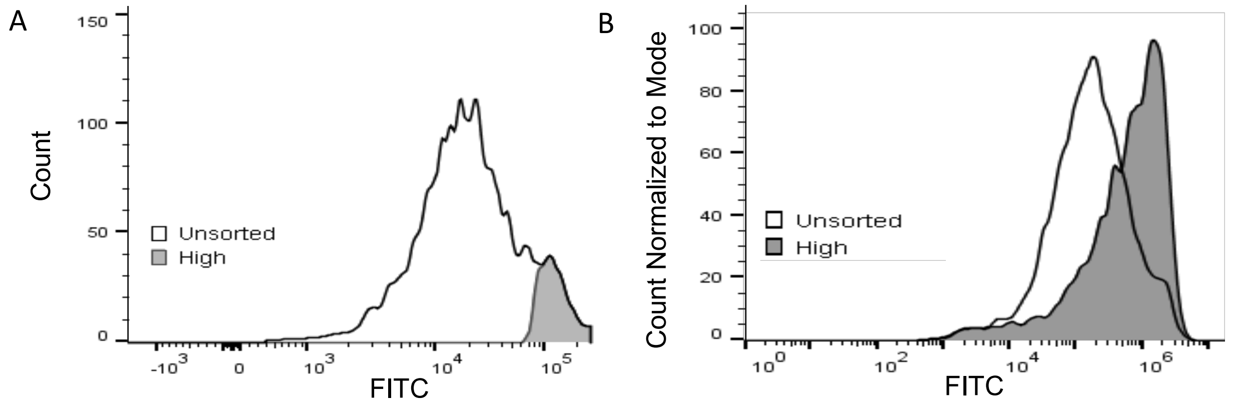

Figure S2. Fluorescent activated cell sorting of high-GD2 KELLY cells. (A) KELLY neuroblastoma cells were evaluated for GD2 expression. Out of the positive population, approximately the top 12% were collected as high-GD2 expressing KELLY cells. (B) Representative Flow cytometry analysis of high expressing GD2 cells implanted into the mouse model as compared to unsorted KELLY cells.

Supplemental Figure 3

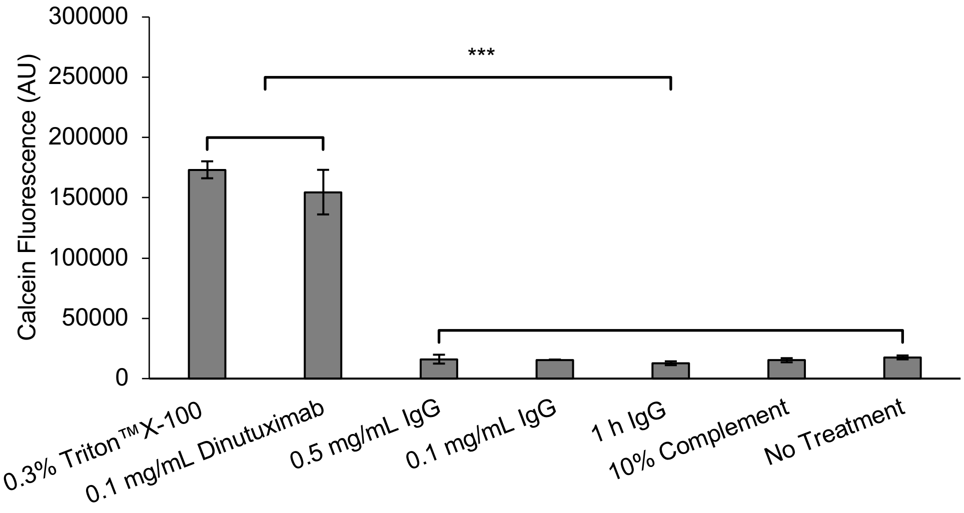

Figure S3. Complement dependent cytotoxicity assay validation. Calcein fluorescence in the supernatant from cells co-treated with 0.1 mg/mL dinutuximab, 0.5 mg/mLIgG, 0.1 mg/mL IgG, or one hour IgG release product and 10% complement serum was compared to 0.3% Triton™ X-100 treatment (0.3% Triton™ X-100), untreated cells (No Treatment) and 10% complement only (10% Complement), *** $p < 0.001$.

Supplemental Figure 4

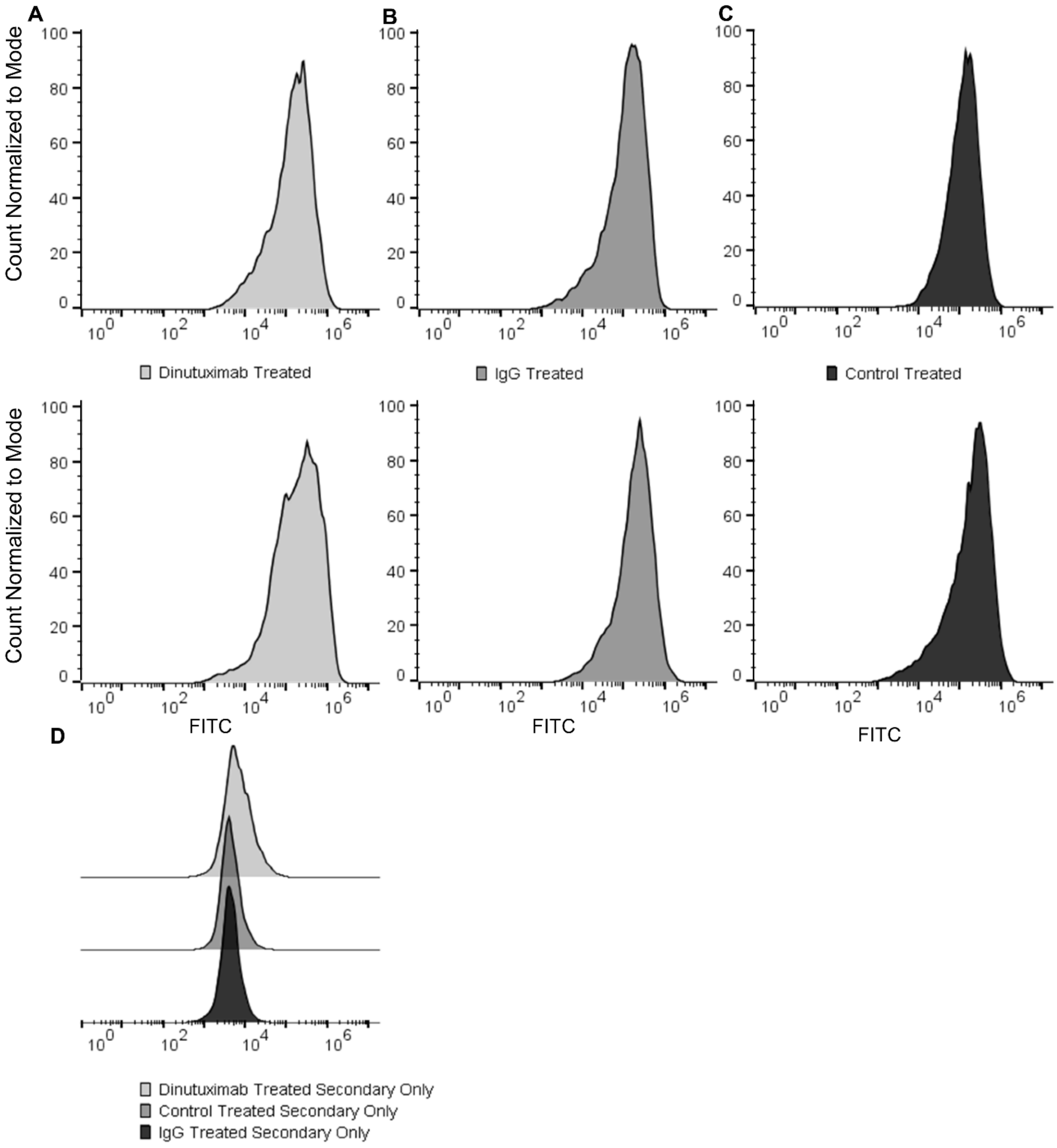

Figure S4. Dissociation tumor cell expression of GD2 after treatment. Dissociated tumor cells were analyzed for GD2 expression using flow cytometry. Representative histograms of GD2 expression as demonstrated by the shift in fluorescent expression are shown for (A) dinutuximab-loaded, (B) IgG-loaded, and (C) control foam treated tumors. (D) Representative histograms of the shift in fluorescent expression of the secondary only control for dinutuximab-loaded, IgG-loaded, and control foam treated tumors.
